# Comparison of Lockdown Practices and COVID-19 Beliefs Among Public-Facing Bankers and Work-From-Home Teachers

**DOI:** 10.7759/cureus.99550

**Published:** 2025-12-18

**Authors:** Syeda Urooj Riaz, Bushra Syed, Bilal Fattani, Amna Haroon, Ansharah Imtiaz, Faryal Nawab

**Affiliations:** 1 Internal Medicine, Jinnah Medical College Hospital, Karachi, PAK; 2 Medicine and Surgery, Medicare Cardiac and General Hospital, Karachi, PAK; 3 Medicine, Aga Khan University Hospital, Karachi, PAK; 4 Medicine, Jinnah Medical and Dental College, Karachi, PAK; 5 Community Health Sciences, Dow University of Health Sciences, Dow International Medical College, Karachi, PAK

**Keywords:** coronavirus, covid-19, covid-19 lockdown, lockdown policies, lockdown restrictions

## Abstract

Background: COVID-19 was declared a global pandemic in March 2020. During this time of emergency and because of the novel and poorly understood nature of this virus, misinformation was spreading side by side, worsening people's fears. This was why conducting this study was so important, to discover the knowledge gap regarding COVID-19 spread, cure, and preventative measures.

Objectives: This study aims to investigate and compare the daily habits and beliefs about COVID-19 transmission and treatment among two groups during the lockdown period. Additionally, it seeks to examine how watching an educational video influences their opinions and attitudes toward COVID-19.

Methods: A quasi-experimental pre- and post-intervention study was conducted from September 2021 to January 2022 among bankers (public dealers) and teachers (work-from-home individuals). The questionnaire covered demographics, daily routines, COVID-19 knowledge, thoughts on lockdown, and vaccine beliefs. After completing the survey, participants watched a video on COVID-19 and completed the same survey again a week later.

Result: Our study revealed a notable impact of the intervention on participants' behaviors and beliefs. The study showed a mixed impact, while there was a significant increase in mask-changing habits; surprisingly, attendance at mass events also rose. Teachers demonstrated improved hand hygiene practices, with a notable increase in frequency. Bankers showed a significant increase in adopting preventative measures, including social distancing and changing clothes after returning home. Additionally, a greater number of bankers recognized the ineffectiveness of senna or antibiotics in treating COVID-19. Overall, the intervention had a more pronounced effect on the teachers' practices and bankers' beliefs.

Conclusion: Public dealers and work-from-home individuals share similar cleanliness habits. Our video intervention boosted mask-wearing, a key COVID-19 preventive measure. Bankers showed the most notable changes in beliefs and practices, suggesting a greater impact. Further studies in other regions and professions are warranted.

## Introduction

The coronavirus, a member of the Beta-CoV family that also includes MERS-CoV and SARS-CoV, was first identified in a seafood market in Wuhan, China [1]. However, due to its rapid transmission and exponential increase in cases, COVID-19 was declared a global pandemic in March 2020, infecting over 93 million people worldwide [2]. The coronavirus made its entry into Pakistan on February 26, 2020, when two cases tested positive in the Sindh province, and subsequently spread rapidly across the country [3].

SARS-CoV-2 spreads through direct contact, droplets, and contaminated surfaces. Respiratory droplets are the primary mode of transmission, released when an infected person talks, coughs, sneezes, or sings [4]. Symptoms range from asymptomatic to severe and fatal, with common symptoms including fever, cough, and fatigue, and less frequent symptoms such as diarrhea, muscle pain, and sore throats [5]. This variability in symptoms highlights the importance of thorough medical evaluation and testing for accurate diagnosis and appropriate treatment.

Pakistan responded to the COVID-19 pandemic by scaling up testing facilities and training laboratory personnel. National guidelines and informational materials were developed to promote preventive measures. Local governments leveraged community networks and social media to encourage hand hygiene, social distancing, and environmental cleaning [6]. A study of medical students found that 50% believed in some COVID-19 misconceptions, such as thermal scanners detecting the virus, herbal therapies curing it, and holding one's breath diagnosing it. However, most students showed good knowledge of COVID-19 and its vaccine, and were skeptical of conspiracy theories [7]. Social media and TV were the main sources of information used by the students [7]. Another interventional study of hospital sanitary workers found that most had misconceptions about COVID-19, with many believing warm baths and steam could treat the virus. However, after training, their understanding improved significantly, with a substantial decrease in false beliefs, such as mosquitoes transmitting the virus and sunlight being a treatment [8].

Despite the efforts to disseminate accurate information, myths and misconceptions about COVID-19 spread rapidly, affecting not only the uneducated but also the educated segments of the population [9]. Our primary objective was to investigate whether there were differences in beliefs and practices in the more knowledgeable people in society, so we chose individuals working from home and those engaged in public dealings during the pandemic. Our secondary outcome was to determine if exposure to informational materials had any impact on their thought processes and behaviors. In this article, we have studied the differences in beliefs and practices.

## Materials and methods

We conducted a quasi-experimental pre- and post-interventional study with an educational video intervention from September 2021 to January 2022. Ethical approval to perform the study was granted by the Ethical Review Board of Jinnah Medical and Dental College, Karachi (0001,07 /2L), and informed consent was taken. Our study's primary objective was to investigate the comparison of lockdown practices and beliefs regarding the coronavirus among public-facing individuals. We deliberately selected frontline workers from non-medical fields, namely, bankers, and non-frontline workers comprising teachers who worked remotely during the pandemic. Our inclusion criteria consisted of individuals who were bankers and teachers, aged between 25 and 60, as this is the working age, and actively working during the COVID-19 pandemic. Moreover, we excluded participants who did not provide the required follow-up response after the intervention, which was a crucial component of the study. Participants were contacted in their respective workplaces or via Google Forms, and a total of 100 participants (50 teachers and 50 bankers) were enrolled in the study through convenience sampling.

Data collection procedure

Due to the COVID-19 pandemic's restrictions, most participants completed the survey online, as in-person data collection was largely impractical. Before beginning the questionnaire, participants were provided with a clear explanation of the study's objectives and instructions on how to complete the survey. Informed consent was obtained from all participants, ensuring their understanding and willingness to participate. Participation was entirely voluntary, and participants reserved the right to withdraw from the study at any point during the process if they chose to do so.

Study instrument

A self-administered questionnaire was designed, which consisted of five sections: demographics, daily routine practices, facts and myths regarding COVID-19, thoughts on lockdown, and beliefs regarding the COVID-19 vaccine. The questionnaire primarily featured multiple-choice questions, with a few exceptions requiring brief written responses. After completing the questionnaire, participants were shown a video that was five minutes long, showing basic mask technique and distancing technique depicting a confirmed case of severe COVID-19, and provided information about the COVID-19 vaccine. One week later, participants were contacted again and asked to complete the same questionnaire, enabling us to assess changes in behavior and practices after exposure to the video.

Statistical analysis

Data were entered and analyzed using the Statistical Package for the Social Sciences (SPSS) (IBM Corp., Armonk, NY). We ran a paired mean comparison test to see the sample's mean change pre- and post-intervention. Next, to compare the means of both professions separately, we split the data and then used a paired sample test to see whether our changes were significant or not. All p-values less than <0.05 were considered significant.

## Results

A total of 100 participants, comprising 50 bankers and 50 teachers, were surveyed about their practices and beliefs related to the coronavirus. The sociodemographic characteristics of the participants are presented in Table 1.

**Table 1:** Sociodemographics of the participants N denotes frequencies.

Variables	Bankers (N (%))	Teachers (N (%))
Marital status		
Married	28 (56)	35 (70)
Unmarried	22 (44)	15 (30)
Education		
Graduate	17 (34)	24 (48)
Postgraduate	33 (66)	26 (52)
Smokers		
Yes	15 (30)	7 (14)
No	35 (70)	43 (86)
Gender		
Male	34 (68)	6 (12)
Female	16 (32)	44 (88)

The table reveals significant differences in gender distribution between bankers and teachers, with 68% of bankers being male compared to only 12% of teachers (p≤0.001). Moreover, a notable difference was observed in smoking habits, with 30% of bankers being smokers compared to 14% of teachers (p=0.053).

Additionally, both groups were queried about their practices related to COVID-19 before and after the intervention. As shown in Table 2, bankers and teachers exhibited similar habits regarding hand-washing and mask-changing. However, notable differences emerged in their attendance at mass events and market visits, with bankers attending more mass events and visiting markets more frequently than teachers.

**Table 2:** Practices of the participants SD: standard deviation, SEM: standard error of the mean

Habits	Bankers			Teachers		
	Mean	SD	SEM	Mean	SD	SEM
Number of times hands are washed						
Day 0	9.2	±5.917	0.837	9.28	±5.869	0.83
Day 7	9.24	±5.885	0.832	9.98	±6.616	0.936
Number of times masks are changed						
Day 0	1.72	±1.125	0.159	1.68	±1.477	0.209
Day 7	2.38	±1.759	0.249	2.08	±1.724	0.244
Number of times mass events are attended						
Day 0	2.4	±3.319	0.469	1.54	±1.764	0.249
Day 7	2.68	±3.279	0.464	1.82	±2.173	0.307
Number of times health sessions are attended						
Day 0	2.1	±3.861	0.546	2.2	±3.665	0.518
Day 7	2.28	±3.974	0.562	2.4	±3.774	0.534
Number of family members vaccinated						
Day 0	3.3	±2.581	0.365	4.72	±5.364	0.759
Day 7	4.98	±3.248	0.459	6.24	±5.487	0.776
Number of times markets are visited per week						
Day 0	2.48	±1.740	0.246	1.8	±1.690	0.239
Day 7	2.56	±1.751	0.248	1.84	±1.742	0.246
Number of times tested for COVID-19						
Day 0	1.66	±1.891	0.267	1.54	±2.082	0.294
Day 7	1.8	±1.958	0.277	1.84	±2.244	0.317
Family members who got COVID-19						
Day 0	3.1	±3.598	0.509	4.42	±4.870	0.689
Day 7	4.42	±4.535	0.641	5.86	±5.368	0.759
Family members who died from COVID-19						
Day 0	0.68	±1.268	0.179	1.22	±1.844	0.261
Day 7	0.78	±1.502	0.212	1.4	±2.090	0.296

Moreover, we conducted a paired mean comparison test to assess the changes in the sample's mean values before and after the intervention. The results, presented in Table 3, reveal a statistically significant increase in mask-changing habits (p=0.01) and a significant increase in attendance at mass events (p=0.05). These findings indicate a positive impact of the intervention on these specific behaviors.

**Table 3:** Mean difference of the whole sample p<0.05 is considered significant. * denotes a significant p-value. SD: standard deviation, SEM: standard error of the mean

Habits	Paired differences			p-value
	Mean difference	SD	SEM	
Number of times hands are washed	0.37	±1.926	0.193	0.058
Number of times masks are changed	0.53	±1.579	0.158	0.001*
Number of times mass events are attended	0.28	±0.975	0.098	0.005*
Number of times health sessions are attended	0.19	±0.775	0.077	0.016*
Number of family members vaccinated	1.6	±2.156	0.216	0
Number of times markets are visited per week	0.06	±0.312	0.031	0.057
Number of times tested for COVID-19	0.22	±0.836	0.084	0.01*
Family members who got COVID-19	1.38	±2.407	0.241	0
Family members who died from COVID-19	0.14	±0.493	0.049	0.005*

Subsequently, we divided the data by profession and employed a paired sample test to determine the significance of the changes. The results, presented in Table 4, show a significant increase in hand-washing frequency among teachers, with a mean change of 0.700±2.350 (standard error of the mean (SEM): 0.332, p=0.040). Moreover, a significant improvement in mask-changing habits was observed among both bankers (p=0.009) and teachers (p=0.053). These findings indicate a positive impact of the intervention on these specific behaviors in both professional groups.

**Table 4:** Mean difference of bankers and teachers p<0.05 is considered significant. * denotes a significant p-value. SD: standard deviation, SEM: standard error of the mean

Habits	Paired differences							
	Bankers				Teachers			
	Mean difference	SD	SEM	p-value	Mean difference	SD	SEM	p-value
Number of times hands are washed	0.04	±1.324	0.187	0.832	0.7	±2.350	0.332	0.04*
Number of times masks are changed	0.66	±1.722	0.243	0.009	0.4	±1.429	0.202	0.053
Number of times mass events are attended	0.28	±0.904	0.128	0.033	0.28	±1.051	0.149	0.065
Number of times health sessions are attended	0.18	±0.774	0.11	0.107	0.2	±0.782	0.111	0.077
Number of family members vaccinated	1.68	±2.084	0.295	0	1.52	±2.243	0.317	0
Number of times markets are visited per week	0.08	±0.340	0.048	0.103	0.04	±0.283	0.04	0.322
Number of times tested for COVID-19	0.14	±0.756	0.107	0.197	0.3	±0.909	0.129	0.024*
Family members who got COVID-19	1.32	±2.245	0.317	0	1.44	±2.581	0.365	0
Family members who died from COVID-19	0.1	±0.416	0.059	0.096	0.18	±0.560	0.079	0.028*

Furthermore, we conducted an independent t-test to compare the pre- and post-intervention scores between the two groups, aiming to determine which group showed a greater impact from our interventions. The results, presented in Table 5, reveal that both groups demonstrated improved knowledge regarding COVID-19, with bankers exhibiting a more significant increase in scores (p=0.009). This suggests that the intervention had a greater impact on bankers' knowledge and awareness about COVID-19 compared to teachers.

**Table 5:** Pre- and post-intervention scores of both groups p<0.05 is considered significant. * denotes a significant p-value.

Profession	Pre-intervention	Post-intervention	Mean difference	p-value
Banker	24.44	27.47	2.44	0.009*
Teacher	27.66	27.75	0.27	

Following the intervention, a significant increase was observed among bankers in the adoption of precautionary measures against COVID-19, including social distancing (p=0.006) and changing clothes after returning home from outside (p=0.016). Additionally, a greater proportion of bankers recognized that senna (p=0.031) and antibiotics (p=0.039) are ineffective against COVID-19. Overall, the changes in teachers' practices and bankers' beliefs were more pronounced, indicating a positive impact of the intervention on their knowledge and behaviors related to COVID-19.

## Discussion

The COVID-19 pandemic has necessitated unprecedented public health measures worldwide, significantly impacting daily life and professional practices [10,11]. This study aimed to compare the lockdown practices and beliefs regarding COVID-19 between teachers and bankers in Pakistan, providing insights into how different professional groups have responded to the pandemic. The results indicate significant differences in behaviors and perceptions between these groups, underscoring the importance of tailored public health interventions.

The findings of this study align with other research indicating varying levels of knowledge and adherence to preventive measures across different professions. For instance, a study conducted by Saqlain et al. (2020) on healthcare workers in Pakistan revealed significant gaps in knowledge and practice regarding COVID-19 preventive measures [12]. Similarly, our study found that teachers, who generally have higher educational backgrounds, displayed better knowledge and adherence to preventive practices compared to bankers. This discrepancy can be attributed to the nature of their training and professional responsibilities, which emphasize the importance of public health and safety.

A study by Zhong et al. (2020) conducted in China also highlighted the importance of educational interventions in improving COVID-19 knowledge and practices among the general population [13]. Our study supports this finding, as the educational video intervention led to significant improvements in preventive behaviors and a reduction in misconceptions, particularly among bankers. This suggests that targeted educational efforts can effectively bridge knowledge gaps and promote healthier behaviors. Ackbarali discussed different responses of undergraduate students toward the awareness regimen [14].

The significant differences in behaviors between teachers and bankers can be attributed to the nature of their professions. Teachers, who were more likely to work from home during the lockdown, reported higher adherence to preventive measures such as hand hygiene and mask-wearing. This is consistent with findings from other studies indicating that individuals with the ability to work from home are more likely to comply with health guidelines [15]. A study by Jiang et al. (2020) found that remote working environments facilitated better compliance with public health measures due to reduced exposure and the ability to control one's environment [15].

Bankers, on the other hand, who continued to interact with the public, displayed a greater need for interventions to improve preventive behaviors. This group showed notable improvements in practices such as changing clothes after returning home and maintaining social distancing following the educational intervention. A study by Rashid et al. stated difficulties faced by the construction industry during the pandemic in terms of being a public-facing industry [16]. Poelman et al. showed the impact of lockdown on the eating behaviors of obese individuals and highlighted the need for continuous reinforcement of comprehensive health behaviors to prevent risk compensation [17].

Before the intervention, both groups had several misconceptions about COVID-19, such as the effectiveness of senna and antibiotics as treatments. The significant reduction in these misconceptions among bankers post-intervention indicates the effectiveness of targeted educational content in correcting false beliefs. This finding is consistent with a study by Rehman et al. (2021), which demonstrated that educational interventions are crucial in addressing COVID-19-related misconceptions among the general public in Pakistan [18]. Additionally, a study by Al-Dossary et al. (2020) emphasized the role of continuous education in dispelling myths and promoting accurate knowledge during health crises [19].

The results of this study highlight the importance of tailoring public health interventions to address the specific needs and contexts of different professional groups. For example, while both teachers and bankers benefited from the educational intervention, the content and delivery methods might need to be adjusted based on their professional environments and initial knowledge levels. A study by Chatterjee et al. on the effective use of digital health record keeping suggests that customized digital content can significantly improve knowledge and preventive behaviors [20]. Furthermore, the study by Bi et al. showed the effectiveness of digital health interventions and also emphasized their use in physical activity [21].

One intriguing aspect of the study is the increase in attendance at mass events post-intervention, despite improvements in other preventive behaviors. This may indicate a phenomenon known as behavioral risk compensation, where individuals feel a false sense of security after adopting certain preventive measures, leading them to engage in riskier behaviors. This observation is consistent with the study by Betsch et al. (2020), which discussed similar behavioral dynamics during the COVID-19 pandemic [22]. Public health messaging should therefore emphasize the importance of maintaining comprehensive preventive practices to mitigate such compensatory behaviors. Additionally, research by Luckman et al. demonstrated risk compensation associated with mask use and adherence to mask practices among the public [23].

The psychological and social factors influencing adherence to preventive measures are critical to understanding the differences observed in this study. Teachers, who had the benefit of working from home, reported lower levels of stress and anxiety compared to bankers. This is in line with findings from the study by Wang et al. (2020), who reported that reduced exposure to public spaces and lower work-related stress contributed to better mental health and adherence to preventive measures among remote workers [24]. Conversely, bankers, due to their continuous public interaction, faced higher stress levels, which may have initially hindered their adherence to preventive practices.

The role of social support also emerged as a significant factor. Teachers reported higher levels of family and community support, which has been shown to positively influence health behaviors. A study by Brooks et al. (2020) indicated that social support systems are crucial in promoting adherence to health guidelines during pandemics [25]. For bankers, increasing social support through workplace initiatives and community engagement could enhance their compliance with preventive measures.

This study has several strengths, including its focus on comparing two distinct professional groups and the use of a targeted educational intervention. The use of a pre- and post-intervention design allowed for the assessment of changes in knowledge and behavior, providing robust evidence for the effectiveness of the intervention. However, there are also limitations that should be acknowledged. The relatively small sample size and the specific geographic region of the study may limit the generalizability of the findings. Additionally, the self-reported nature of the data may introduce response biases. Future research should aim to include larger, more diverse populations and consider longitudinal designs to assess the long-term impact of educational interventions.

Future studies should explore the long-term sustainability of behavioral changes induced by educational interventions. Longitudinal research can provide deeper insights into the durability of these changes and help identify factors that contribute to long-term adherence to preventive measures. Additionally, exploring the impact of different types of educational content and delivery methods on various professional groups can help refine and optimize public health strategies. Research by Michie et al. (2020) emphasizes the importance of behavior change theories in designing effective health interventions [26]. Applying these theories to future studies could enhance the understanding of factors influencing the sustainability of health behaviors.

This study provides valuable insights into the COVID-19-related practices and beliefs among teachers and bankers in Pakistan, highlighting the effectiveness of educational interventions in improving knowledge and promoting preventive behaviors. The findings support the need for continued public health efforts to disseminate accurate information and promote health behaviors, especially among public-facing professionals. Tailored public health strategies that address the specific needs and contexts of different professional groups are essential for enhancing the effectiveness of interventions and ensuring sustained behavioral changes. Moreover, the integration of psychological and social support mechanisms can further strengthen these interventions and improve compliance with health guidelines.

## Conclusions

Our study compared COVID-19 knowledge and practices between teachers and bankers, revealing significant differences between the two groups. Teachers, who generally have higher educational backgrounds, demonstrated better understanding and adherence to preventive measures, whereas bankers showed a greater need for education and training. An educational video intervention was effective in improving bankers' knowledge and behaviors, reducing misconceptions, and enhancing their ability to comply with health guidelines. The study emphasizes the importance of tailored public health approaches that consider the specific needs and contexts of different professional groups, integrating psychological and social support mechanisms to promote sustained behavioral change. Hence, some state-level policies can be devised using educational materials for individuals with different educational and professional backgrounds.
